# Classroom climate and EFL students’ willingness to communicate: the mediating role of L2 grit

**DOI:** 10.3389/fpsyg.2026.1808101

**Published:** 2026-07-16

**Authors:** Didem Erdel

**Affiliations:** Department of Western Languages and Literatures, Faculty of Science and Letters, Igdir University, Iğdır, Türkiye

**Keywords:** classroom climate, classroom environment, L2 grit, L2 WTC, willingness to communicate

## Abstract

**Introduction:**

This study investigated the relationships among classroom social climate, L2 willingness to communicate (WTC) and L2 grit, with particular attention to the mediating role of L2 grit, in a Turkish EFL context. Drawing on socio-contextual and intrapersonal perspectives on L2 learning, the study aimed to bridge interpersonal classroom dynamics and learner-internal characteristics within a single mediation framework.

**Methods:**

Data were collected from 227 undergraduate EFL learners using self-report measures of perceived classroom climate, L2 grit, and L2 WTC. Structural equation modeling was employed to examine the hypothesized relationships, and mediation was further tested using bootstrap procedures.

**Results:**

The results indicated that classroom social climate had a significant positive direct effect on L2 WTC and also exerted an indirect effect through L2 grit. Classroom social climate significantly predicted L2 grit, which in turn emerged as a strong predictor of L2 WTC. The significant direct and indirect effects provided evidence for a partial mediating role of L2 grit in the relationship between classroom social climate and WTC.

**Discussion:**

These findings suggest that supportive classroom climates promote learners’ willingness to communicate both directly and by strengthening their L2 grit, highlighting the interplay between contextual and learner-internal factors in L2 communication. The study offers pedagogical implications for fostering supportive learning environments and sustaining learner engagement.

## 1 Introduction

Foreign/Second language (L2) learning is a complex and multifaceted process in which intrapersonal, interpersonal, emotional, and cognitive dimensions are deeply intertwined. For several decades, L2 research was predominantly cognition-oriented, prioritizing the examination of intellectual processes involved in language learning (Prior, 2019). More recently, however, there has been growing recognition of the pivotal role of social and individual factors in shaping L2 development. From both sociocultural and social-cognitive perspectives, language learning is fundamentally a social endeavor in which learners’ interactions with their surrounding environment are central to the learning process (Bandura, 1986; Lantolf, 2006). Accordingly, the classroom, as the primary instructional context, can be argued to exert a substantial influence on language learning outcomes.

This emphasis on contextual influence aligns closely with Bronfenbrenner’s (1979) ecological view of human development, which underscores the role of the classroom environment, alongside cognitive and affective factors, in shaping students’ academic success. When learners experience a strong sense of interpersonal connection within the classroom, more advanced social and relational skills tend to emerge (Nixon and Patrick, 2024). Supportive and nurturing classroom climates have been shown to foster higher levels of self-esteem and cognitive growth (Zedan, 2010). Similarly, positive social environments facilitate interactions and activities that promote L2 learners’ psychological well-being (Joe et al., 2017). In contrast, classrooms characterized by a weaker emotional climate often display strained teacher–student relationships, diminished mutual respect, and instructional practices that are insufficiently responsive to learners’ perspectives, cognitive needs, or affective states such as boredom, discomfort, and confusion (Reyes et al., 2012).

Beyond interpersonal dynamics, intrapersonal characteristics have also been widely acknowledged as critical determinants of L2 development. The second construct addressed in the present study, willingness to communicate in a second/foreign language (L2 WTC), concerns learners’ exercise of linguistic agency as they negotiate the choice between verbal participation and communicative withdrawal (Barabadi et al., 2023). A well-documented paradox in L2 classrooms is the presence of learners who, despite recognizing the necessity of communication for language development, remain reluctant to engage orally in the target language (Peng, 2015). Conceptualized as the final psychological threshold preceding actual speech, WTC is widely regarded as a key driving force behind L2 interaction. Such interaction is of particular importance, as it enables access to pedagogical opportunities and interpersonal exchanges that would otherwise remain unavailable within formal instructional settings (Peng, 2015).

Language learning is inherently a long-term endeavor that requires sustained effort and enduring passion (Botes et al., 2025; Khajavy et al., 2021; Mikami, 2024), qualities that are central to the construct of “grit” (Duckworth and Quinn, 2009). Achievement in language acquisition often depends on learners’ capacity to persist and maintain interest in learning tasks despite encountering linguistic or pedagogical challenges (Khajavy and Aghaee, 2024). In this respect, grit–particularly L2 grit as a domain-specific construct reflecting learners’ perseverance in language learning (Sudina and Plonsky, 2021)–has been conceptualized as a non-cognitive factor (Khajavy et al., 2021) that contributes meaningfully to the attainment of educational goals (Cheng and Cui, 2026; Xu and Hong, 2025; Pawlak et al., 2024). Learners who demonstrate sustained passion and perseverance in language learning are more likely to achieve higher levels of success, display stronger motivation, engage more actively in classroom activities (Botes et al., 2025), and pursue long-term objectives consistently, maintaining effort irrespective of immediate feedback or external reinforcement (Duckworth et al., 2007).

Within the Turkish educational context, EFL classrooms are frequently characterized by a pronounced tendency toward communicative avoidance among learners (Başöz and Erten, 2019). This pattern underscores the need to examine not only contextual influences such as classroom social climate but also the intrapersonal mechanisms through which these influences may shape learners’ communicative behavior. Current research in this cultural context highlights several determinants, including classroom components (teaching practices, classroom atmosphere, class size) and affective factors (Darıyemez and Yastıbaş, 2023; Ölmez and İlter, 2025; Salbaş and Ekmekçi, 2025; Solhi and Thumvichit, 2025), as well as positive psychological constructs such as savoring beliefs (Soyoof et al., 2025). Accordingly, the present study aims to inspect L2 classroom social climate, students’ L2 WTC, and L2 grit using a correlational approach, thereby bridging contextual and intrapersonal perspectives on L2 learning and examining the mediating role of L2 grit in the relationship between classroom climate and L2 WTC.

### 1.1 Literature review

#### 1.1.1 Classroom social climate

Classroom social climate refers to the dynamic qualities of a classroom that extend beyond its physical characteristics to encompass socioecological and socioemotional dimensions, including teachers’ and students’ attitudes, behaviors, actions, and interactions (Derakhshan et al., 2024; Dörnyei and Murphey, 2003; Hosseini et al., 2022). These dynamics involve interpersonal relationships, the emotional tone of classroom interactions, and structural features of instructional practices and classroom organization, as well as teachers’ expectations of and attitudes toward students, degrees of teacher control, disciplinary practices, and learner characteristics such as age and gender (Zedan, 2010).

A widely cited conceptualization of classroom social climate is offered by Patrick et al. (2007), who proposed a four-component model comprising teacher support, student support, classroom mutual respect, and task-related interaction. In a subsequent refinement of the model, Patrick et al. (2011) differentiated teacher support into academic and emotional dimensions and removed the explicit nominalization of student support, while retaining the core relational emphasis of the framework.

Learners’ perceptions of the classroom environment play a pivotal role in shaping their self-beliefs and interpretations of schoolwork, which in turn influence both the quality and intensity of academic engagement (Patrick et al., 2007). A positive classroom climate has been associated with enhanced EFL learning motivation (Fraser and Fisher, 1982), greater use of self-regulation strategies (Alzubaidi et al., 2016; Patrick et al., 2007), higher levels of student engagement (Derakhshan et al., 2024; Hosseini et al., 2022; Li, 2024; Ma and Wei, 2022; Wang et al., 2025; Yang and Liang, 2025), improved retention (Dwyer et al., 2004), stronger academic performance (Ma and Wei, 2022; Reyes et al., 2012), and more positive learner emotions such as foreign language enjoyment (Hosseini et al., 2022; Wang and Wang, 2024), trait emotional intelligence (Wang and Wang, 2024) and academic resilience (Wang and Patterson, 2025). Beyond these academic and affective outcomes, classroom climate has also been shown to contribute to the establishment of a secure learning environment, the development of clear behavioral norms, the reduction of violent tendencies, and the promotion of students’ socio-emotional development (Sağkal et al., 2015).

Within this expanding literature, increasing attention has been directed toward the relationship between classroom social climate and L2 WTC. Joe et al. (2017) showed that a positive classroom climate predicted learners’ basic psychological need satisfaction, which in turn influenced WTC. Khajavy et al. (2018) found that a positive classroom environment promoted enjoyment and WTC while reducing anxiety. Similarly, Wang et al. (2021) demonstrated that classroom social climate influenced L2 WTC indirectly through learners’ academic emotions, including enjoyment, pride, anxiety, and boredom. Given that WTC represents a critical behavioral manifestation of learners’ engagement and agency in language use, a closer examination of L2 WTC is warranted.

#### 1.1.2 L2 WTC

Willingness to communicate functions as an immediate precursor to communicative action and occupies a central position in the second/foreign language acquisition process (Lan et al., 2021). It is commonly defined as “an individual’s volitional inclination toward actively engaging in the act of communication in a specific situation, which can vary according to interlocutor, topic, and conversational context, among other potential situational variables” (Kang, 2005, p. 291). Since its formal introduction to the L2 literature by MacIntyre et al. (1998), WTC has attracted sustained scholarly attention and has been regarded as one of the most desirable learner characteristics, given its strong association with successful language learning outcomes (Henry and MacIntyre, 2023).

MacIntyre et al. (1998) conceptualized WTC through a heuristic pyramid model in which multiple layers of influence, ranging from enduring factors such as personality and intergroup climate to more immediate situational processes, converge to shape learners’ WTC. Later, MacIntyre (2020) argued that this model overlooks intra-individual variability and the moment-to-moment fluctuations in WTC driven by changing classroom conditions, interlocutors, emotions, and task demands, reflecting a shift toward viewing WTC as an emergent, context-dependent state rather than a stable trait. Building on this dynamic perspective, it has been argued that L2 WTC research should focus on identifying the variables that contribute to fluctuations in learners’ WTC (Henry and MacIntyre, 2023). From this viewpoint, WTC emerges from the continuous interaction between internal learner attributes and external pedagogical influences, particularly those embedded in the teacher-mediated classroom environment (Dewaele and Dewaele, 2018; MacIntyre, 2020).

A substantial body of research has documented the role of individual and affective factors in shaping L2 WTC. These include ideal L2 self (Lan et al., 2021), positive and negative L2 emotions such as enjoyment and anxiety (Dewaele and Pavelescu, 2021), perceived communicative competence, motivation, self-confidence, and anxiety (Başöz and Erten, 2019; Elahi Shirvan et al., 2019; Sak, 2020; Teimouri et al., 2019; Wang and Cionea, 2024), perfectionist cognition (Barabadi et al., 2023), openness to experience (Piechurska-Kuciel, 2018), emotional intelligence (Wang and Xu, 2026), personality traits (Dewaele and Pavelescu, 2021; Oz, 2014), learners’ attitudes toward the L2 and social L2 enjoyment (Dewaele and Dewaele, 2018), as well as teaching practices and classroom atmosphere (Başöz and Erten, 2019; Dewaele and Dewaele, 2018). In addition to correlational evidence, intervention-based research has demonstrated that L2 WTC is amenable to pedagogical influence. For example, instructional interventions incorporating motivational strategies have been shown to exert long-term positive effects on learners’ L2 WTC (Ye and Hu, 2025). Similarly, academic engagement training significantly enhanced students’ L2 WTC (Zeng and Chen, 2026). These findings further support the view of WTC as a malleable construct responsive to instructional and contextual manipulation.

Because L2 WTC cannot be fully explained by individual-level variables such as language competence or learner beliefs alone, growing attention has been directed toward classroom social climate as a critical contextual determinant (Cao, 2014; Mystkowska-Wiertelak, 2021; Wang et al., 2021). Empirical evidence suggests that even minor changes in communicative contexts can rapidly alter learners’ affective states, shifting them from WTC to reluctance or withdrawal (Yashima et al., 2018). Peng (2012) further characterized L2 WTC as “socioculturally constructed,” demonstrating that it is shaped by classroom environment alongside individual, cognitive, linguistic, and affective factors. Further supporting this view, Li et al. (2025) reported significant associations between classroom environment and WTC, while Wang et al. (2024) found that classroom social climate indirectly influenced WTC through positive emotions and language mindset.

More recently, research has begun to examine the relationship between L2 WTC and L2 grit, the third construct addressed in the present study. Chen et al. (2025) demonstrated both direct and indirect effects of L2 grit on EFL learners’ WTC, with positive psychological resources–namely optimism, resilience, efficacy, and hope–serving as mediators. Similarly, Rashid and Malik (2026) reported a strong and significant effect of L2 grit on L2 WTC, mediated by L2 enjoyment. These findings suggest that perseverance and sustained effort in language learning may constitute an important intrapersonal mechanism underlying learners’ willingness to engage in L2 communication.

#### 1.1.3 L2 grit

Over the past few decades, positive personality traits have attracted increasing scholarly attention, particularly under the influence of positive psychology in L2 pedagogy. Positive psychology aims “to understand, test, discover and promote the factors that allow individuals and communities to thrive” (Sheldon et al., 2000, p. 1), and its integration into L2 research has marked a paradigmatic shift from a predominant focus on deficits and negative emotions toward learners’ strengths and positive capacities (MacIntyre, 2016). Within this framework, grit, defined as “perseverance and passion for long-term goals” (Duckworth et al., 2007, p. 1087), has emerged as a salient positive character trait (Dewaele et al., 2019). Despite the growing interest it has received over the past decade, grit–particularly in its domain-specific L2 form–remains a relatively underexplored construct in second language acquisition research (Elahi Shirvan et al., 2022; Zou et al., 2025).

Conceptually, grit comprises two interrelated components: perseverance of effort, referring to the capacity to maintain effort against long-term challenges, and consistency of interest, which denotes the ability to preserve stable interest in a goal over time (Alamer, 2021; Duckworth et al., 2021). Learners with high consistency of interest are better able to sustain enthusiasm despite obstacles, whereas those exhibiting strong perseverance of effort tend to persist and continue working through setbacks and periods of stagnation (Khajavy et al., 2025). Importantly, although grit has occasionally been conflated with related constructs such as resilience, self-control, or conscientiousness, it is theoretically distinct in its emphasis on long-term goal commitment and sustained passion rather than short-term regulation or situational coping (Pawlak et al., 2024).

Within L2 learning contexts, grit has been increasingly recognized as a meaningful non-cognitive factor contributing to language development (Khajavy et al., 2025). Empirical research has linked grit to higher academic performance in both general education and L2 contexts (Duckworth et al., 2007; Harpaz et al., 2024; Mikami, 2024; Teimouri et al., 2022a,b). More specifically, Zou et al. (2025) demonstrated that L2 grit played a crucial role in academic achievement, which in turn significantly enhanced academic engagement. Similarly, Botes et al. (2025) found that L2 grit served as a moderate predictor of academic performance and a strong predictor of student engagement and motivation. Supporting these findings, Pawlak et al. (2024) reported that both L2 grit and general grit predicted L2 motivation.

Beyond direct effects, a growing body of research has highlighted the mediating and facilitative roles of L2 grit within broader motivational and affective frameworks. Chen et al. (2021) showed that L2 grit functioned as a psychological intermediary linking motivation to specific L2 skill outcomes, enhancing self-efficacy while reducing language anxiety. Xu and Hong (2025) likewise reported that grit played a key mediating role in the relationship between emotional intelligence and L2 learning performance. In relation to learner self-concepts, Khajavy et al. (2021) found that a growth language mindset weakly but positively predicted perseverance of effort, whereas a fixed mindset negatively predicted consistency of interest, underscoring the differential susceptibility of grit components to cognitive orientations.

Importantly, recent studies have drawn attention to the componential and context-dependent nature of L2 grit. Khajavy and Aghaee (2024) reported that perseverance of effort was associated with L2 emotions and personal best goals and indirectly related to L2 achievement, suggesting a limited and situational role for consistency of interest in predicting language outcomes. Similarly, Zhang (2023) demonstrated that perseverance of effort predicted and mediated the effects of writing achievement goals on L2 writing performance. Comparing L2 grit with aptitude, Teimouri et al. (2022b) concluded that grit could serve as a stronger predictor of L2 achievement, further emphasizing its explanatory potential in SLA. Nevertheless, Khajavy et al. (2025) found only a small correlation between L2 grit and conscientiousness, lending support to grit’s partial distinctiveness from broader personality traits.

Despite its growing prominence, grit has not been without criticism. Concerns have been raised regarding its conceptual distinctiveness, arguing that it substantially overlaps with established personality constructs such as conscientiousness, and the construct validity of measurement instruments used to assess it as meta-analytic evidence has suggested that grit may be only a modest predictor of academic achievement and that its two dimensions do not contribute equally to educational outcomes, with perseverance of effort consistently demonstrating stronger predictive power than consistency of interest (Credé et al., 2017). Nevertheless, accumulating evidence supports its divergent validity as an independent construct, particularly in educational and L2-specific contexts, suggesting that its effects cannot be fully understood outside the environments in which it is enacted. As existing research remains fragmented, with inconsistent findings across contexts and grit components, a need for more nuanced and integrative analyses of how grit operates within the complex ecology of second language learning emerges (Zou et al., 2025).

### 1.2 The present study

**FIGURE 1:**
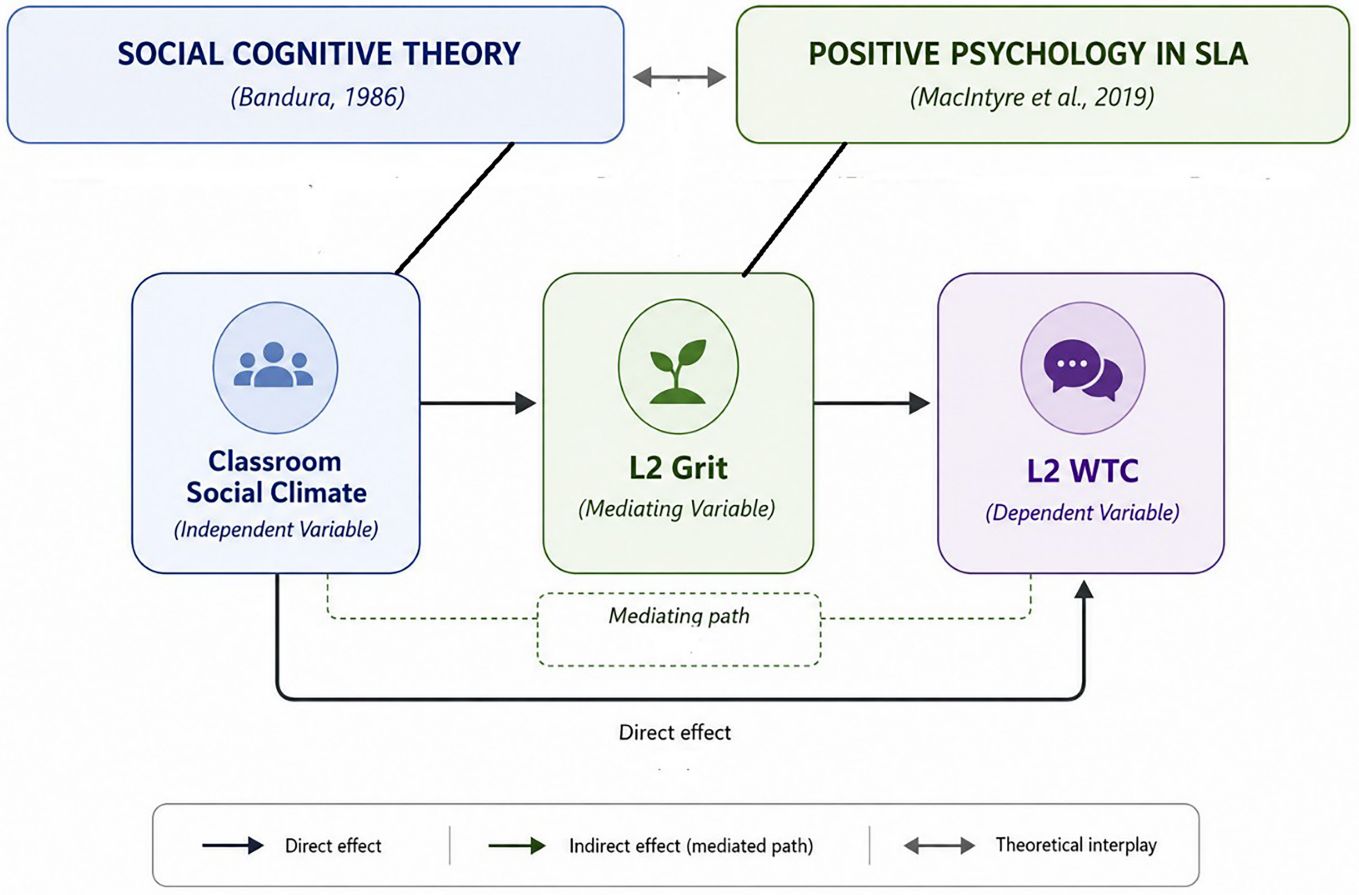
Theoretical framework of the study.

The present study is theoretically grounded in Bandura’s (1986) Social Cognitive Theory and the Positive Psychology perspective in second language acquisition (SLA) (MacIntyre et al., 2019). From a social cognitive standpoint, L2 WTC is shaped through reciprocal interactions among personal and linguistic factors, and environmental influences, with classroom social climate constituting a critical contextual determinant (Cao, 2014). Supportive classroom environments characterized by teacher and peer support, mutual respect, and meaningful interaction are likely to enhance learners’ self-beliefs, emotional security, and perceived communicative competence, thereby fostering greater L2 WTC. Complementing this view, positive psychology in SLA emphasizes learners’ positive psychological resources–such as grit–as key mechanisms through which favorable learning environments translate into sustained engagement and communicative behavior. Within this framework, L2 grit is conceptualized as a malleable personal strength that can be nurtured by positive classroom experiences. Accordingly, a supportive classroom social climate is expected to facilitate the development of L2 grit, which in turn strengthens learners’ persistence in communicative attempts and their willingness to use the L2 despite challenges. Figure 1 represents an illustration of the theoretical framework of the present study.

Previous research has examined the relationships among the three constructs addressed in the present study–L2 grit, L2 WTC, and L2 classroom social climate–in connection with various other variables. However, to the best of our knowledge, the interrelationship among L2 grit, L2 WTC, and L2 classroom social climate has not yet been empirically investigated. From a cultural perspective, research focusing on these constructs within Turkish educational settings remains limited and unevenly distributed. Existing studies are largely concentrated on L2 WTC (Başöz and Erten, 2019; Erdem and Alav, 2023; Karadağ and Kaya, 2019; Oz, 2014; Öz et al., 2015; Sak, 2020), whereas empirical work on L2 grit (Derakhshan et al., 2025; Solhi et al., 2025) and L2 classroom social climate (Sak, 2019) is comparatively scarce. Consequently, the construct of L2 grit and its associations with other pedagogical variables in L2 learning have remained largely underexplored in the Turkish context. Similarly, the influence of classroom social environment on learners’ L2 WTC has yet to be sufficiently verified within this cultural setting. The present study therefore, seeks to address this gap by testing the cultural applicability of the hypothesized relationships among these constructs.

At the same time, L2 WTC is widely acknowledged as a context-sensitive construct that may vary across cultural settings (Lee et al., 2022). Informed by recent situated approaches to L2 WTC, the present study examines the interplay between situational and individual determinants of learners’ WTC in formal instructional contexts, focusing specifically on classroom social climate and L2 grit. Accordingly, the purpose of the current study is to elucidate the mechanisms linking classroom social climate, L2 grit, and L2 WTC. The following hypotheses represent the overarching propositions tested in the present study:

1. Classroom social climate is expected to be positively associated with learners’ L2 grit.

2. Learners’ L2 grit is expected to be positively related to their L2 WTC.

3. Classroom social climate is expected to positively associate with L2 WTC with a mediating role of L2 grit.

## 2 Materials and methods

### 2.1 Research design

This is a single-mediation correlational study. Correlation studies involve “procedures in quantitative research in which investigators measure the degree of association (or relation) between two or more variables using the statistical procedure of correlational analysis” (Creswell, 2012, p. 21). Given that the present study sought to investigate the magnitude and direction of the relationships among classroom social climate, L2 grit, and willingness to communicate, as well as to test a theoretically grounded mediation model, a correlational design provided an appropriate framework for addressing the research questions while preserving the naturally occurring nature of the variables under investigation.

### 2.2 Participants and procedure

The research participants consisted of students enrolled in the English Language and Literature department at a state university in eastern Türkiye. The department offers a 5-years curriculum, beginning with a 1-year compulsory English preparatory program. A total population sampling was employed as the research population was small and manageable. All students enrolled in the department at the time of data collection (approximately *N* = 250) were invited to participate in the study. Consequently, the data collection instrument was administered to all classes within the department. A total of 227 valid responses were obtained, which exceeded commonly recommended minimum sample sizes for structural equation modeling and was considered adequate for the complexity of the proposed model. The participant group comprised preparatory students (*N* = 45), freshmen (*N* = 44), sophomores (*N* = 47), juniors (*N* = 42), and seniors (*N* = 46); three students did not specify their year of study. Regarding gender distribution, the participants included 164 females and 49 males, with 11 missing responses and 3 “I do not want to share” responses.

Before data collection, ethical approval was obtained from the university’s ethical review board. The research data were collected through paper-based questionnaires. The students were informed about the purpose and scope of the study and were assured of anonymity and voluntary participation before the administration of data collection.

### 2.3 Data collection instruments

#### 2.3.1 Connected Classroom Climate Inventory

To ensure linguistic and conceptual equivalence, the Turkish version of the Connected Classroom Climate Inventory (Dwyer et al., 2004), adapted and validated by Sağkal et al. (2015), was employed to collect data on participants’ perceptions of the social climate of their classrooms. The scale is a one-factor instrument consisting of 18 items, rated on a 5-point Likert scale ranging from 1 (strongly disagree) to 5 (strongly agree). The reliability coefficient of the scale was reported as 0.94 in the original version (Dwyer et al., 2004) and 0.93 in the Turkish adaptation (Sağkal et al., 2015). Moreover, the instrument has been used and validated across diverse academic and cultural contexts (Johnson, 2009; MacLeod et al., 2018; Nixon and Patrick, 2024). In the present study, the Cronbach’s alpha coefficient was found 0.93, which indicates high reliability for the scale.

#### 2.3.2 L2 Grit Scale

Originally developed for Persian EFL learners, the L2 Grit Scale (Teimouri et al., 2022a) was deemed the most appropriate instrument for the present study due to its domain-specific focus. Accordingly, the Turkish adaptation of the scale (Uştuk and Erarslan, 2023) was employed. Both the original and Turkish versions comprise two subscales–persistence of effort and consistency of interest–with a total of nine items. Responses are recorded on a 5-point Likert scale ranging from “not like me at all” to “very much like me.” The scale has demonstrated satisfactory validity and reliability in both its original and adapted forms. Moreover, the L2 Grit Scale has been used and validated across diverse cultural contexts, including Polish (Pawlak et al., 2024), Chinese (Chen et al., 2025) and Japanese (Mikami, 2024) settings. In the Turkish adaptation, Cronbach’s alpha coefficients ranged from 0.84 to 0.90 for the overall scale and its subscales, and the validity of the two-factor, nine-item structure was confirmed through exploratory factor analysis (Uştuk and Erarslan, 2023).

For the present study, initial reliability and validity analyses indicated that one item (Item 2) from the consistency of interest subscale exhibited a very low standardized factor loading (0.17) and a weak item–total correlation (0.19), suggesting an inadequate contribution to the construct; therefore, this item was removed from the analysis. In addition, one item (item 9) from the perseverance of effort subscale in the original scale was inadvertently omitted during data collection. Although these modifications resulted in a version of the instrument that differed slightly from the original scale, the underlying two-factor structure of L2 grit was retained since despite this omission, the remaining items demonstrated strong corrected item–total correlations (0.51–0.66) and satisfactory internal consistency. The reliability coefficients (Cronbach’s alpha) were.83 for the overall scale and 0.78 and 0.86 for the perseverance of effort and consistency of interest subscales, respectively, indicating that the instrument exhibited acceptable reliability. Consequently, the scale was retained in a seven-item, two-factor structure for the analyses. Accordingly, the results should be interpreted as pertaining to this version of the L2 Grit Scale, which may somewhat limit direct comparability with studies employing the original instrument.

#### 2.3.3 Willingness to communicate scale

Students’ WTC was measured using the Turkish adaptation (Öksüz Zerey and Cephe, 2020) of the L2 Willingness to Communicate Questionnaire originally developed by Weaver (2005). The original instrument consists of 17 items in a single-factor structure, whereas the Turkish version comprises 15 items, with two items removed due to concerns related to construct validity. In both versions, responses are recorded on a 4-point Likert-type scale (1 = definitely not willing; 2 = probably not willing; 3 = probably willing; 4 = definitely willing). The adapted scale demonstrated satisfactory internal consistency (α = 0.87), and its construct validity was supported by exploratory factor analysis, with the 15-item structure explaining 35.888% of the total variance (Öksüz Zerey and Cephe, 2020). In the present study, the reliability coefficient (Cronbach’s alpha) was 0.90, suggesting strong internal consistency of the scale.

It should be noted that the instruments utilized in the study employed different response formats, with two scales using a 5-point Likert format and one scale using a 4-point Likert format. However, this difference is unlikely to have materially affected the findings because, as described in detail in the “Results,” the constructs were modeled as latent variables within the SEM framework. Moreover, each scale demonstrated satisfactory psychometric properties, and the measurement and structural models exhibited acceptable fit, suggesting that the different response formats did not compromise the validity of the results.

### 2.4 Data analysis

The data were first examined for normality by inspecting Skewness and Kurtosis values, as well as Q–Q plots and histograms, to assess the suitability of the data for subsequent parametric analyses. The internal consistency reliability of the scales used in the study was assessed using Cronbach’s alpha. To evaluate the potential presence of common method bias, Harman’s single-factor test was conducted by entering all items from the three scales into an unrotated exploratory factor analysis. Pearson product–moment bivariate correlations were then computed to examine the relationships among the three constructs of the study. In addition, the effects of gender and school year on students’ responses to the scales were tested using an independent-samples *t*-test and one-way ANOVA, respectively, as these variables comprised two-group and multi-group comparisons.

Subsequently, the measurement model was tested through confirmatory factor analysis (CFA) using AMOS 26 to establish the validity of the latent constructs and their observed indicators. The CFA included the latent variables of classroom climate, L2 grit (comprising the dimensions of perseverance of effort and consistency of interest), and WTC, along with their observed indicators. Finally, the hypothesized structural model was tested using structural equation modeling (SEM), which enables the simultaneous examination of direct and indirect relationships among latent variables. In this model, classroom climate was specified as the exogenous variable, whereas L2 grit and WTC were treated as endogenous variables. To corroborate the mediation effects estimated in the SEM framework, a robust bootstrap-based mediation analysis was performed using the PROCESS macro (Model 4) with 5,000 resamples and 95% confidence intervals (Hayes, 2022), as bootstrap procedures are recommended for assessing the significance of indirect effects.

## 3 Results

### 3.1 Common method bias

The results showed that the first factor accounted for 25% of the total variance, which is below the 50% threshold, indicating that common method bias was not a serious concern in this study (Podsakoff et al., 2024).

### 3.2 Descriptive overview and correlations of the study variables

The normal distribution of data was confirmed with the Skewness and Kurtosis values, which were acceptable in range (±1) (Tabachnick and Fidell, 2013), and visual inspection of the histograms and Q–Q plots confirmed the distribution was approximately normal, as the histograms exhibited a bell-shaped curve and the data points in the Q–Q plots adhered to the diagonal reference line.

As Table 1 shows, all three constructs were in moderate but statistically significant correlation. As hypothesized, classroom climate was positively associated with both L2 grit (*r* = 0.14, *p* < 0.05) and WTC (*r* = 0.23, *p* < 0.001). Although the magnitude of these relationships was small, the statistically significant correlations may suggest that more positive perceptions of the classroom climate tend to co-occur with higher levels of grit and a greater WTC in the L2. In addition, L2 grit was moderately and positively correlated with WTC (*r* = 0.41, *p* < 0.001), providing preliminary support for the proposed mediating role of L2 grit in the relationship between classroom climate and WTC.

**TABLE 1:** Descriptive statistics and correlations.

Scales	*M*	*SD*	Skewness	Kurtosis	1	2	3
1. Classroom climate	2.987	0.781	−0.386	−0.166	1	1	1
2. L2 grit	3.654	0.747	−0.635	0.014	0.140*		
3. Willingness to communicate	2.974	0.620	−0.693	0.689	0.226***	0.411***	

*p < 0.05; ***p < 0.001.

### 3.3 Test of the measurement model

**FIGURE 2:**
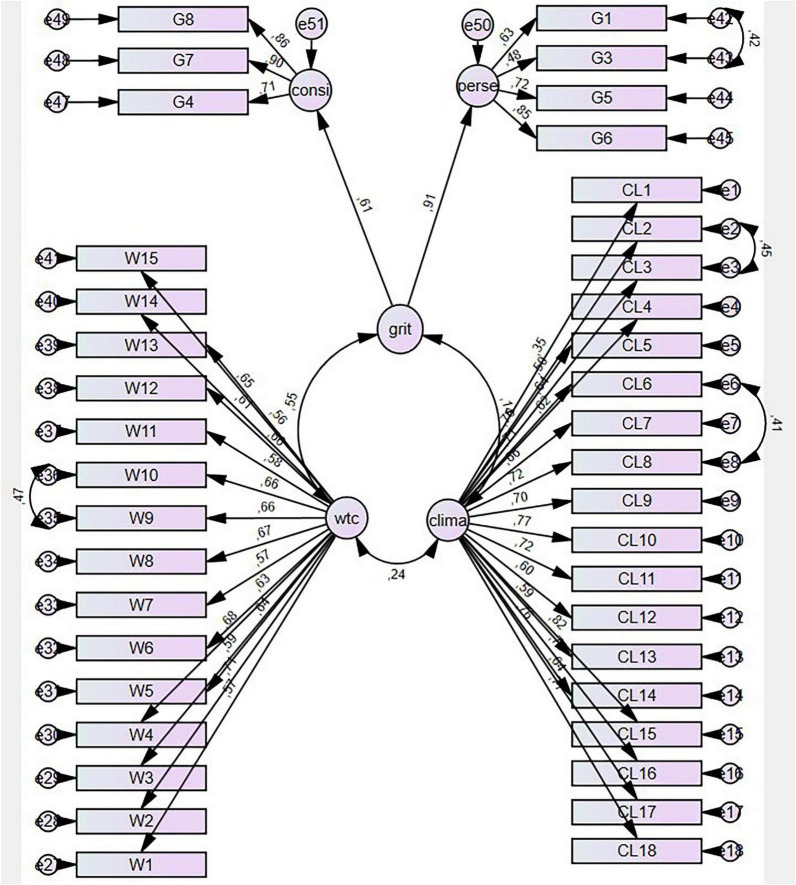
Measurement model.

The discriminant validity of the three primary latent variables was examined through CFA, with the measurement model specified by 40 observed indicators (see Figure 2). A small number of error covariances were added between items with similar wording and content, guided by modification indices and theoretical plausibility. Similar covariances were observed in original validation studies as well (Sağkal et al., 2015). The model fit results were as follows: χ^2^/df = 1.692; CFI = 0.889; TLI = 0.882; RMSEA = 0.055. Given the sensitivity of CFI and TLI to the number of indicators and sample size, the complexity and item count of the present model suggest that the observed CFI (0.89) and TLI (0.88) are acceptable when interpreted contextually rather than against universal cutoff values (Marsh et al., 2004). SRMR was also calculated using lavaan (Rosseel, 2012) in R Statistical Software (v4.5.2; R Core Team, 2025) to supplement the fit indices obtained from AMOS, as this index was not available in the AMOS output. The value indicated an acceptable model fit (SRMR = 0.07). Eventually, the SRMR and RMSEA values were within acceptable ranges, and χ^2^/df indicating a good model fit provided support for an adequate overall model fit.

As indicated in Figure 2, standardized regression weights of items in all three scales were above 0.50 excluding one item (Item 1 in Classroom Social Climate Scale) which displayed a relatively lower standardized loading (0.35); however, it was retained as it was considered to exceed the minimum acceptable threshold, and the internal consistency results demonstrated an acceptable item–total correlation, and no negative effect on scale reliability.

### 3.4 Structural equation modeling of the proposed mediation model

Preliminary analyses were conducted to examine potential differences across key demographic variables. A one-way ANOVA revealed a significant difference across academic levels for perceived classroom social climate, *F*(4, 219) = 5.07, *p* < 0.001, whereas no significant differences were observed for L2 grit or WTC (*p* > 0.05). As expected, this pattern suggests that classroom social climate is more sensitive to contextual variations across academic levels, while L2 grit and WTC function as relatively stable learner characteristics. In addition, preliminary independent samples *t*-tests indicated no significant gender differences across any of the study variables. Given that neither academic level nor gender was associated with the mediator or outcome variable, these demographic factors were not included in the subsequent structural equation modeling.

**FIGURE 3:**
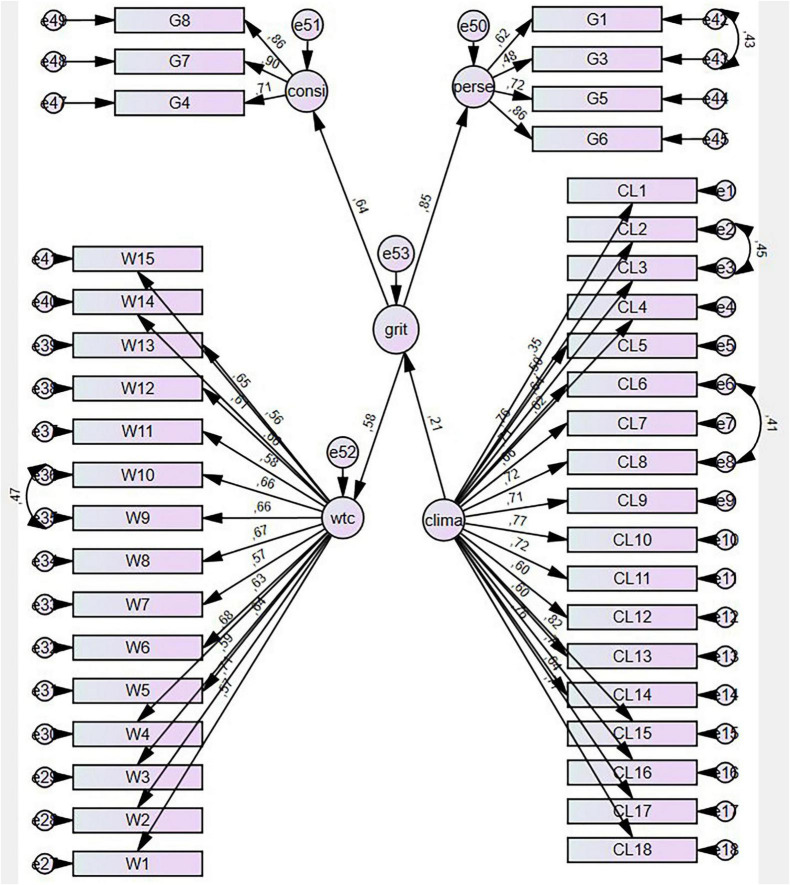
Path diagram of the hypothesized mediation model.

The structural equation model showed an acceptable fit to the data, χ^2^/df = 1.698, CFI = 0.888, TLI = 0.882, RMSEA = 0.056, 90% CI [0.050, 0.061], and SRMR = 0.07. Although the CFI and TLI values were marginal–likely reflecting model complexity and item count as discussed in the measurement model–the absolute fit indices indicated satisfactory model fit. The path diagram and standardized path coefficients (β) are shown in Figure 3.

Structural equation modeling results indicated that classroom social climate had a positive direct effect on WTC (β = 0.14, *p* = 0.005), while also exerting an indirect effect through L2 grit. The bootstrap mediation analysis conducted using PROCESS with 5,000 resamples further supported this finding, revealing a significant indirect effect of classroom social climate on WTC via L2 grit as the 95% bootstrap confidence interval did not include zero (indirect effect = 0.043, BootSE = 0.020, 95% bootstrap CI [0.006, 0.087]). In addition, classroom social climate significantly predicted L2 grit (β = 0.15, 95% CI [0.008, 0.285]), and L2 grit was a significant predictor of WTC (β = 0.29, 95% CI [0.192, 0.391]). Since both the direct and indirect effects remained significant, the results provide evidence for a partial mediating role of L2 grit in the relationship between classroom social climate and WTC.

## 4 Discussion

This paper presents a correlational account of the effect of L2 classroom social climate on ELF students’ WTC through L2 grit in a mediating role. The results have confirmed the hypothesized correlations and mediation. While the effect sizes were modest, they remain meaningful given the complexity of classroom interactional settings and the multifaceted nature of WTC. Even small effects of classroom social climate may exert a cumulative influence on learners’ motivational dispositions over time, highlighting the practical relevance of supportive classroom environments.

Regarding the first hypothesis, the findings indicated that the classroom social climate was a predictor of L2 grit. Apparently, students’ perseverance and interest in L2 learning thrived as the interpersonal qualities such as trust, respect, kindness, and support among classmates increased. The results receive support from earlier literature demonstrating the significant effects of teacher-student and peer relationships on the development of L2 grit (Cui and Yang, 2022). Empirical evidence indicates that perceived support and care from teachers and peer rapport enable EFL learners to cope more effectively with learning challenges, fostering greater hope for goal attainment and promoting the development of higher levels of L2 grit (Yuan, 2022). Nonetheless, it should be acknowledged that other contextual and individual factors may also contribute to the relationships observed in the present study. For example, classroom-related variables such as teacher characteristics and instructional practices, as well as learner variables such as motivation, anxiety or personality traits, may influence the development of L2 grit and students’ perceptions of classroom climate. However, because these factors were not examined in the present study, any discussion of their specific effects would be speculative. In addition, year of study was found to be associated with students’ perceptions of classroom climate, suggesting that learners at different stages of their university education may experience the classroom environment differently.

As regards the second hypothesis, significant L2 grit influence on L2 WTC was confirmed. The findings were parallel with earlier studies (Lan et al., 2021; Lee, 2022; Rashid and Malik, 2026; Wang et al., 2022). Previous research similarly suggests that possessing more positive psychological resources significantly elevates a learner’s WTC in the target language (Chen et al., 2025). Apparently, learners who exhibit higher levels of L2 grit tend to invest greater effort in the language learning process, persist in the face of communicative challenges, and maintain sustained motivation to develop their linguistic competence, and therefore potentially have higher WTC (Derakhshan and Fathi, 2025; Lee and Drajati, 2019).

The third hypothesis of the study, proposing the effect of classroom social climate on L2 WTC with the mediation of L2 grit, was also confirmed by the results. First, the results indicated that classroom social climate played both a direct and an indirect role in shaping EFL learners’ WTC. The significant positive direct effect of classroom social climate on L2 WTC suggests that learners who perceive their classroom environment as supportive, respectful, and rich in interaction are more inclined to engage in L2 communication. Earlier research lends credence to these results (Altiner, 2022; Darıyemez and Yastıbaş, 2023; Li et al., 2025; Wang et al., 2021, 2024). Secondly and very importantly, the findings also demonstrated that L2 grit played a mediating role in classroom climate and L2 WTC relationship. The results imply that higher L2 WTC is possible with L2 grit as long as a positive classroom climate is established, as classroom social climate was found to exert an indirect effect on L2 WTC. Teachers, therefore, should find ways of creating facilitative learning environments and avoid debilitative circumstances from occurring, since while individual learner profiles remain largely fixed, teachers possess the agency to strategically manipulate a wide array of contextual variables (Mystkowska-Wiertelak, 2021).

## 5 Conclusion

In practical terms, the results suggest that efforts to enhance learners’ WTC should not be limited to modifying classroom interactional patterns alone. In this sense, classroom climate and L2 grit operate synergistically rather than independently in promoting L2 WTC. While a supportive classroom climate may foster opportunities for interaction and emotional safety, learners’ willingness to capitalize on these affordances may depend on enduring individual characteristics, such as their capacity for sustained effort and perseverance in language learning. Hence, creating a positive classroom social climate remains essential; nevertheless, fostering learners’ perseverance and long-term engagement with language learning may amplify the impact of such environments on communicative outcomes.

The findings of the present study highlight the importance of creating supportive, positive, and facilitative learning environments in which long-term learning goals can be pursued without discouraging learners’ sustained effort and interest. Classrooms characterized by respect and emotional safety are particularly important, as learners are less likely to lose their WTC when their perseverance and interest in language learning are maintained. In this regard, instructional practices should aim to minimize demotivating competitive pressures while fostering conditions that enable students to remain committed to their learning goals.

A further pedagogical implication concerns the importance of fostering learners’ commitment to long-term educational aspirations while simultaneously cultivating a supportive and psychologically secure classroom climate. Such an environment may help mitigate debilitating experiences, sustain learners’ perseverance in the face of challenges, and promote more adaptive engagement with the language learning process. Extracurricular initiatives may also play a valuable role in fostering a positive social climate. One possible approach would be to encourage students to form departmental learning communities that facilitate interaction and collaboration across cohorts, thereby promoting mutual support, collective growth, and sustained engagement. Teachers should promote classroom cultures by incorporating collaborative learning activities such as group projects, presentations or demonstrations and peer assessment more frequently, thereby normalizing the processes of giving and receiving constructive feedback. Establishing feedback practices that are self-referential, normative, and promotion-oriented can contribute to sustaining learners’ WTC by reinforcing perseverance and continued engagement in L2 interaction (Zarrinabadi and Dehkordi, 2024).

As regards theoretical implications, the present study contributes to the L2 learning literature by bridging interpersonal and intrapersonal dimensions through a mediation model that has not previously been documented, thereby offering a more integrative account of how classroom social climate and learner-internal resources jointly shape WTC. It must also be noted that although the study is conducted in the Turkish EFL context, the proposed model is derived from broader theoretical frameworks of WTC, classroom climate, and L2 grit. Therefore, the relationships examined are not assumed to be unique to Turkish learners, though it is acknowledged that the applicability of the model is likely to be susceptible to educational and cultural factors.

Despite its contributions, the study is subject to certain limitations. The exclusive reliance on self-report scales based on students’ perceptions may have constrained the objectivity of the findings, and the context-specific nature of the sample raises concerns regarding the generalizability of the results to other educational and cultural settings. Future research may test the model in different teaching and learning contexts. It may also benefit from employing more complex analytical approaches, such as extended or chain mediation models, to further disentangle the dynamic and multi-layered mechanisms through which contextual and individual factors interact in L2 learning.

## Data Availability

The raw data supporting the conclusions of this article will be made available by the author, without undue reservation.

## References

[B1] Alamer A. (2021). Grit and language learning: construct validation of L2-Grit scale and its relation to later vocabulary knowledge. *Educ. Psychol.* 41 544–562. 10.1080/01443410.2020.1867076

[B2] Altiner C. (2022). Effect of classroom environment on Turkish EFL learners’ willingness to communicate. *Pesa Uluslararası Sosyal Araştırmalar Dergisi* 8 130–140. 10.25272/j.2149-8385.2022.8.3.03

[B3] Alzubaidi E. Aldridge J. M. Khine M. S. (2016). Learning English as a second language at the university level in Jordan: motivation, self-regulation and learning environment perceptions. *Learn. Environ. Res.* 19 133–152. 10.1007/s10984-014-9169-7

[B4] Bandura A. (1986). *Social Foundations of Thought and Action: A Social Cognitive Theory.* Hoboken, NJ: Prentice- Hall.

[B5] Barabadi E. Khajavy G. H. Booth J. R. Rahmani Tabar M. Vahdani Asadi M. R. (2023). The links between perfectionistic cognitions, L2 achievement and willingness to communicate: examining L2 anxiety as a mediator. *Curr. Psychol.* 42 30878–30890. 10.1007/s12144-022-04114-7

[B6] Başöz T. Erten I. H. (2019). A qualitative inquiry into the factors influencing EFL learners’ in-class willingness to communicate in English. *Novitas-ROYAL* 13 1–18. Available online at: https://eric.ed.gov/?id=EJ1214141

[B7] Botes E. Azari Noughabi M. Amirian S. M. R. Greiff S. (2025). New wine in new bottles? L2 Grit in comparison to domain-general grit, conscientiousness, and cognitive ability as a predictor of language learning. *J. Multilingual Multicult. Dev.* 46 2451–2466. 10.1080/01434632.2023.2294120

[B8] Bronfenbrenner U. (1979). *The Ecology of Human Development.* Cambridge, MA: Harvard University Press.

[B9] Cao Y. (2014). A sociocognitive perspective on second language classroom willingness to communicate. *Tesol Q.* 48 789–814. 10.1002/tesq.155

[B10] Chen X. Alruwaili A. R. Azari Noughabi M. Ghasemi A. Zhen C. (2025). The mediating role of psychological capital in the relationship between EFL learners’ L2 grit and L2 WTC. *Front. Psychol.* 16:1621340. 10.3389/fpsyg.2025.1621340 40709236 PMC12288664

[B11] Chen X. Lake J. Padilla A. M. (2021). Grit and motivation for learning English among Japanese university students. *System* 96:102411. 10.1016/j.system.2020.102411

[B12] Cheng E. H. Cui T. (2026). Is L2 grit a hierarchical construct? A meta-analysis targeting language learners. *J. Multiling. Multicult. Dev.* 47 357–375. 10.1080/01434632.2024.2390568

[B13] Credé M. Tynan M. C. Harms P. D. (2017). Much ado about grit: a meta-analytic synthesis of the grit literature. *J. Pers. Soc. Psychol.* 113:492. 10.1037/pspp0000102 27845531

[B14] Creswell J. W. (2012). *Educational Research: Planning, Conducting, and Evaluating Quantitative and Qualitative Research.* London: Pearson.

[B15] Cui T. Yang Y. (2022). Social relationships and grit in English as a foreign language learning among high school students: a three-wave longitudinal study. *Front. Psychol.* 13:1038878. 10.3389/fpsyg.2022.1038878 36262446 PMC9574368

[B16] Darıyemez T. Yastıbaş A. E. (2023). EFL students’ suggestions to maintain their willingness to communicate in online English language lessons. *GIST–Educ. Learn. Res. J.* 26 7–27. 10.26817/16925777.1615

[B17] Derakhshan A. Fathi J. (2025). Longitudinal exploration of interconnectedness through a cross-lagged panel design: enjoyment, anxiety, willingness to communicate, and L2 grit in English language learning. *J. Multiling. Multicult. Dev.* 46 2192–2210. 10.1080/01434632.2024.2393705

[B18] Derakhshan A. Fathi J. Pawlak M. Kruk M. (2024). Classroom social climate, growth language mindset, and student engagement: the mediating role of boredom in learning English as a foreign language. *J. Multiling. Multicult. Dev.* 45 3415–3433. 10.1080/01434632.2022.2099407

[B19] Derakhshan A. Solhi M. Azari Noughabi M. (2025). An investigation into the association between student-perceived affective teacher variables and students’ L2-grit. *J. Multiling. Multicult. Dev.* 46 798–814. 10.1080/01434632.2023.2212644

[B20] Dewaele J. M. Chen X. Padilla A. M. Lake J. (2019). The flowering of positive psychology in foreign language teaching and acquisition research. *Front. Psychol.* 10:2128. 10.3389/fpsyg.2019.02128 31607981 PMC6769100

[B21] Dewaele J. M. Dewaele L. (2018). Learner-internal and learner-external predictors of willingness to communicate in the FL classroom. *J. Eur. Sec. Lang. Assoc.* 2 24–37. 10.22599/jesla.37

[B22] Dewaele J. M. Pavelescu L. M. (2021). The relationship between incommensurable emotions and willingness to communicate in English as a foreign language: a multiple case study. *Innovat. Lang. Learn. Teach.* 15 66–80. 10.1080/17501229.2019.1675667

[B23] Dörnyei Z. Murphey T. (2003). *Group Dynamics in the Language Classroom.* Cambridge: Cambridge University Press.

[B24] Duckworth A. L. Peterson C. Matthews M. D. Kelly D. R. (2007). Grit: perseverance and passion for long-term goals. *J. Pers. Soc. Psychol.* 92 1087–1101. 10.1037/0022-3514.92.6.1087 17547490

[B25] Duckworth A. L. Quinn P. D. (2009). Development and validation of the short grit scale (Grit-S). *J. Pers. Assess.* 91 166–174. 10.1080/00223890802634290 19205937

[B26] Duckworth A. L. Quinn P. D. Tsukayama E. (2021). Revisiting the factor structure of grit: a commentary on Duckworth and Quinn (2009). *J. Pers. Assess.* 103 573–575. 10.1080/00223891.2021.1942022 34254861

[B27] Dwyer K. K. Bingham S. G. Carlson R. E. Prisbell M. Cruz A. M. Fus D. A. (2004). Communication and connectedness in the classroom: development of the connected classroom climate inventory. *Commun. Res. Rep.* 21 264–272. 10.1080/08824090409359988

[B28] Elahi Shirvan M. Khajavy G. H. MacIntyre P. D. Taherian T. (2019). A meta-analysis of L2 willingness to communicate and its three high-evidence correlates. *J. Psycholinguist. Res.* 48 1241–1267. 10.1007/s10936-019-09656-9 31342240

[B29] Elahi Shirvan M. Taherian T. Yazdanmehr E. (2022). L2 grit: a longitudinal confirmatory factor analysis-curve of factors model. *Stud. Sec. Lang. Acquisit.* 44 1449–1476. 10.1017/S0272263121000590

[B30] Erdem A. T. Alav O. (2023). İletişim İsteksizliği Ölçeği: Türkçeye Uyarlama, Geçerlik ve Güvenirlik Çalışması [The Unwillingness-to-Communicate: the study of adaptation to Turkish, validity and reliability]. *Abant Sosyal Bilimler Dergisi* 23 1387–1404. 10.11616/asbi.1327859

[B31] Fraser B. J. Fisher D. L. (1982). Predicting student outcomes from their perceptions of classroom psychosocial environment. *Am. Educ. Res. J.* 19 498–518. Available online at: https://www.jstor.org/stable/1162539

[B32] Harpaz G. Vaizman T. Yaffe Y. (2024). University students’ academic grit and academic achievements predicted by subjective well-being, coping resources, and self-cultivation characteristics. *High. Educ. Q.* 78 192–211. 10.1111/hequ.12455

[B33] Hayes A. F. (2022). *Introduction to Mediation, Moderation, and Conditional Process Analysis: A Regression-Based Approach*, 3rd Edn. New York, NY: Guilford Publications.

[B34] Henry A. MacIntyre P. D. (2023). *Willingness to Communicate, Multilingualism and Interactions in Community Contexts.* Bristol: Multilingual Matters.

[B35] Hosseini H. M. Fathi J. Derakhshesh A. Mehraein S. (2022). A model of classroom social climate, foreign language enjoyment, and student engagement among English as a foreign language learners. *Front. Psychol.* 13:933842. 10.3389/fpsyg.2022.933842 36059776 PMC9428561

[B36] Joe H. K. Hiver P. Al-Hoorie A. H. (2017). Classroom social climate, self-determined motivation, willingness to communicate, and achievement: a study of structural relationships in instructed second language settings. *Learn. Individ. Differ.* 53 133–144. 10.1016/j.lindif.2016.11.005

[B37] Johnson D. I. (2009). Connected classroom climate: a validity study. *Commun. Res. Rep.* 26 146–157. 10.1080/08824090902861622

[B38] Kang S. J. (2005). Dynamic emergence of situational willingness to communicate in a second language. *System* 33 277–292. 10.1016/j.system.2004.10.004

[B39] Karadağ Ş Kaya S. D. (2019). The effects of personality traits on willingness to communicate: a study on university students. *MANAS Sosyal Araştırmalar Dergisi* 8 397–410. 10.33206/mjss.519145

[B40] Khajavy G. H. Aghaee E. (2024). The contribution of grit, emotions and personal bests to foreign language learning. *J. Multiling. Multicult. Dev.* 45 2300–2314. 10.1080/01434632.2022.2047192

[B41] Khajavy G. H. MacIntyre P. D. Barabadi E. (2018). Role of the emotions and classroom environment in willingness to communicate: applying doubly latent multilevel analysis in second language acquisition research. *Stud. Sec. Lang. Acquisit.* 40 605–624. 10.1017/S0272263117000304

[B42] Khajavy G. H. MacIntyre P. D. Hariri J. (2021). A closer look at grit and language mindset as predictors of foreign language achievement. *Stud. Sec. Lang. Acquisit.* 43 379–402. 10.1017/S0272263120000480

[B43] Khajavy G. H. Modarresi G. Hejazi S. Y. (2025). Exploring and addressing concerns surrounding L2 grit: a longitudinal perspective. *Learn. Individ. Differ.* 119:102660. 10.1016/j.lindif.2025.102660

[B44] Lan G. Nikitina L. Woo W. S. (2021). Ideal L2 self and willingness to communicate: a moderated mediation model of shyness and grit. *System* 99:102503. 10.1016/j.system.2021.102503

[B45] Lantolf J. P. (2006). Sociocultural theory and L2: state of the art. *Stud. Sec. Lang. Acquisit.* 28 67–109. 10.1017/S0272263106060037

[B46] Lee J. S. (2022). The role of grit and classroom enjoyment in EFL learners’ willingness to communicate. *J. Multiling. Multicult. Dev.* 43 452–468. 10.1080/01434632.2020.1746319

[B47] Lee J. S. Drajati N. A. (2019). Affective variables and informal digital learning of English: Keys to willingness to communicate in a second language. *Aust. J. Educ. Technol.* 35 168–182. 10.14742/ajet.5177

[B48] Lee J. S. Lee K. Chen Hsieh J. (2022). Understanding willingness to communicate in L2 between Korean and Taiwanese students. *Lang. Teach. Res.* 26 455–476. 10.1177/1362168819890825

[B49] Li C. Dewaele J. M. Pawlak M. Kruk M. (2025). Classroom environment and willingness to communicate in English: the mediating role of emotions experienced by university students in China. *Lang. Teach. Res.* 29 2140–2160. 10.1177/13621688221111623

[B50] Li M. (2024). Modeling the role of rapport and classroom climate in EMI students’ classroom engagement. *Acta Psychol.* 245:104209. 10.1016/j.actpsy.2024.104209 38513401

[B51] Ma Y. Wei C. (2022). The relationship between perceived classroom climate and academic performance among English-major teacher education students in Guangxi, China: the mediating role of student engagement. *Front. Psychol.* 13:939661. 10.3389/fpsyg.2022.939661 35992415 PMC9387542

[B52] MacIntyre P. (2020). Expanding the theoretical base for the dynamics of willingness to communicate. *Stud. Sec. Lang. Learn. Teach.* 10 111–131. 10.14746/ssllt.2020.10.1.6

[B53] MacIntyre P. D. (2016). “So far so good: an overview of positive psychology and its contributions to SLA,” in *Positive Psychology Perspectives on Foreign Language Learning and Teaching*, eds Gabryś-Barker D. Gałajda D. (Cham: Springer), 3–20.

[B54] MacIntyre P. D. Clément R. Dörnyei Z. Noels K. A. (1998). Conceptualizing willingness to communicate in a L2: a situational model of L2 confidence and affiliation. *Modern Lang. J.* 82 545–562. 10.1111/j.1540-4781.1998.tb05543.x

[B55] MacIntyre P. D. Gregersen T. Mercer S. (2019). Setting an agenda for positive psychology in SLA: theory, practice, and research. *Modern Lang. J.* 103 262–274. 10.1111/modl.12544

[B56] MacLeod J. Yang H. H. Zhu S. Shi Y. (2018). Technological factors and student-to-student connected classroom climate in cloud classrooms. *J. Educ. Comput. Res.* 56 826–847. 10.1177/0735633117733999

[B57] Marsh H. W. Hau K. T. Wen Z. (2004). In search of golden rules: comment on hypothesis-testing approaches to setting cutoff values for fit indexes and dangers in overgeneralizing Hu and Bentler’s (1999) findings. *Struct. Equat. Model.* 11 320–341. 10.1207/s15328007sem1103_2 42420623

[B58] Mikami H. (2024). Revalidation of the L2-Grit scale: a conceptual replication of Teimouri, Y., Plonsky, L., & Tabandeh, F.(2022). L2 grit: passion and perseverance for second-language learning. *Lang. Teach.* 57 274–289. 10.1017/S0261444822000544

[B59] Mystkowska-Wiertelak A. (2021). Fluctuations in willingness to communicate during a semester: a case study. *Lang. Learn. J.* 49 1–12. 10.1080/09571736.2018.1469660

[B60] Nixon G. Patrick C. J. (2024). Validation of the Connected Classroom Climate Inventory in post-graduate health science degrees and the relationship with clinical self-efficacy. *Researchsquare* 10.21203/rs.3.rs-5658533/v1 36284789

[B61] Öksüz Zerey M. Cephe P. T. (2020). An investigation into the relationship between willingness to communicate and classroom environment in a Turkish EFL context. *J. Lang. Linguist. Stud.* 16, 896–911. 10.17263/jlls.759338

[B62] Ölmez G. N. İlter B. G. (2025). Predictors of foreign language speaking anxiety in a tertiary level EFL context. *Porta Linguarum An Int. J. For. Lang. Teach. Learn.* 43 295–310. 10.30827/portalin.vi43.28720

[B63] Oz H. (2014). Big five personality traits and willingness to communicate among foreign language learners in Turkey. *Soc. Behav. Pers.* 42 1473–1482. 10.2224/sbp.2014.42.9.1473

[B64] Öz H. Demirezen M. Pourfeiz J. (2015). Willingness to communicate of EFL learners in Turkish context. *Learn. Individ. Differ.* 37 269–275. 10.1016/j.lindif.2014.12.009

[B65] Patrick H. Kaplan A. Ryan A. (2011). Positive classroom motivational environments: convergence between mastery goal structure and classroom social climate. *J. Educ. Psychol.* 103 367–382. 10.1037/a0023311

[B66] Patrick H. Ryan A. Kaplan A. (2007). Early adolescents’ perceptions of the classroom social environment, motivational beliefs, and engagement. *J. Educ. Psychol.* 99 83–98. 10.1037/0022-0663.99.1.83

[B67] Pawlak M. Chengchen L. Zawodniak J. Kruk M. (2024). Examining predictive effects of general grit and L2 grit on motivated behavior: the mediating effect of self-perceived proficiency. *Porta Linguarum* 11 93–112. 10.30827/portalin.viIX.29884

[B68] Peng J. E. (2012). Towards an ecological understanding of willingness to communicate in EFL classrooms in China. *System* 40 203–213. 10.1016/j.system.2012.02.002

[B69] Peng J. E. (2015). L2 motivational self system, attitudes, and affect as predictors of L2 WTC: an imagined community perspective. *Asia-Pac. Educ. Res.* 24 433–443. 10.1007/s40299-014-0195-0

[B70] Piechurska-Kuciel E. (2018). Openness to experience as a predictor of L2 WTC. *System* 72 190–200. 10.1016/j.system.2018.01.001

[B71] Podsakoff P. M. Podsakoff N. P. Williams L. J. Huang C. Yang J. (2024). Common method bias: it’s bad, it’s complex, it’s widespread, and it’s not easy to fix. *Annu. Rev. Organ. Psychol. Organ. Behav.* 11 17–61. 10.1146/annurev-orgpsych-110721-040030

[B72] Prior M. T. (2019). Elephants in the room: An “affective turn,” or just feeling our way? *Modern Lang. J.* 103 516–527. 10.1111/modl.12573

[B73] R Core Team (2025). *R: A Language and Environment for Statistical Computing (Version 4.5.2) [Computer software].* Vienna: R Foundation for Statistical Computing.

[B74] Rashid S. Malik S. (2026). Foreign language enjoyment, L2 grit, and perceived teacher support in TESOL contexts: a structural equation modeling study of L2 willingness to communicate. *Educ. Sci.* 16:89. 10.3390/educsci16010089

[B75] Reyes M. R. Brackett M. A. Rivers S. E. White M. Salovey P. (2012). Classroom emotional climate, student engagement, and academic achievement. *J. Educ. Psychol.* 104:700. 10.1037/a0027268

[B76] Rosseel Y. (2012). Lavaan: an R package for structural equation modeling. *J. Stat. Softw.* 48 1–36. 10.18637/jss.v048.i02

[B77] Sağkal A. S. Kabasakal Z. T. Türnüklü A. (2015). Turkish adaptation of the connected classroom climate inventory (CCCI). *Element. Educ. Online* 14 1179–1192. 10.17051/io.2015.30422

[B78] Sak M. (2019). Contextual factors that enhance and impair directed motivational currents in instructed L2 classroom settings. *Novitas-Royal* 13 155–174. Available online at: https://eric.ed.gov/?id=EJ1231981

[B79] Sak M. (2020). The role of ideal L2 self in predicting L2 willingness to communicate inside and outside the classroom. *Eur. J. Appl. Linguist.* 6 189–203. 10.32601/ejal.775798

[B80] Salbaş H. Ekmekçi E. (2025). The impact of classroom environment on students’ willingness to communicate in foreign language learning. *Int. J. Educ. Res.* 129:102517. 10.1016/j.ijer.2024.102517

[B81] Sheldon K. Frederikson B. Rathunde K. Csikszentmihalyi M. Haidt J. (2000). “Positive psychology manifesto,” in *Manifest Presented at the Akumal 1 meeting (1999) and revised at the Akumal 2 meeting (2000*, (Mexico).

[B82] Solhi M. Derakhshan A. Ünsal B. (2025). Associations between EFL students’ L2 grit, boredom coping strategies, and emotion regulation strategies: a structural equation modeling approach. *J. Multiling. Multicult. Dev.* 46 224–243. 10.1080/01434632.2023.2175834

[B83] Solhi M. Thumvichit A. (2025). Dissecting subjective L2 (un) willingness to communicate among EFL learners: a Q methodology study. *J. Multiling. Multicult. Dev.* 46 2211–2226. 10.1080/01434632.2024.2349802

[B84] Soyoof A. Azari Noughabi M. Ghasemi A. Solhi M. Leon Liu G. (2025). Exploring the mediating role of savoring beliefs on EFL learners’ informal digital learning of English and willingness to communicate: a cross-cultural perspective. *Innovat. Lang. Learn. Teach.* 1–22. 10.1080/17501229.2025.2554862

[B85] Sudina E. Plonsky L. (2021). Academic perseverance in foreign language learning: an investigation of language-specific grit and its conceptual correlates. *Modern Lang. J.* 105 829–857. 10.1111/modl.12738

[B86] Tabachnick B. G. Fidell L. S. (2013). *Using Multivariate Statistics*, 6th Edn. London: Pearson.

[B87] Teimouri Y. Goetze J. Plonsky L. (2019). Second language anxiety and achievement: a meta-analysis – Erratum. *Stud. Sec. Lang. Acquisit.* 41 489–489. 10.1017/S0272263119000445

[B88] Teimouri Y. Plonsky L. Tabandeh F. (2022a). L2 grit: passion and perseverance for second-language learning. *Lang. Teach. Res.* 26 893–918. 10.1177/1362168820921895

[B89] Teimouri Y. Tabandeh F. Tahmouresi S. (2022b). The hare and the tortoise: the race on the course of L2 learning. *Modern Lang. J.* 106 764–783. 10.1111/modl.12806

[B90] Uştuk Ö Erarslan A. (2023). Adaptation and initial validation of the L2-grit scale in Turkish. *Nevşehir Hacı Bektaş Veli Üniversitesi SBE Dergisi* 13 1176–1188. 10.30783/nevsosbilen.1276339

[B91] Wang H. Patterson M. M. (2025). Academic resilience in learning English as a foreign language: Relations to classroom climate and beliefs about language learning. *Stud. Educ. Eval.* 87:101517. 10.1016/j.stueduc.2025.101517

[B92] Wang H. Patterson M. M. Peng A. (2024). Predictors of second language willingness to communicate among US undergraduate students: classroom social climate, emotions, and language mindset. *Lang. Teach. Res.* 13621688241237214. 10.1177/13621688241237214

[B93] Wang H. Peng A. Patterson M. M. (2021). The roles of class social climate, language mindset, and emotions in predicting willingness to communicate in a foreign language. *System* 99:102529. 10.1016/j.system.2021.102529

[B94] Wang M. Wang H. Shi Y. (2022). The role of English as a foreign language learners’ grit and foreign language anxiety in their willingness to communicate: theoretical perspectives. *Front. Psychol.* 13:1002562. 10.3389/fpsyg.2022.1002562 36186361 PMC9516278

[B95] Wang M. Wang Y. (2024). A structural equation modeling approach in examining EFL students’ foreign language enjoyment, trait emotional intelligence, and classroom climate. *Learn. Motiv.* 86:101981. 10.1016/j.lmot.2024.101981

[B96] Wang R. Xu Q. (2026). Emotional intelligence and language learning performance of EFL learners in China: chain mediating effects of willingness to communicate and foreign language learning boredom. *Front. Psychol.* 16:1702948. 10.3389/fpsyg.2025.1702948 41608187 PMC12835198

[B97] Wang X. Gao Y. Wang Q. Zhang P. (2025). Fostering engagement in AI-mediate Chinese EFL classrooms: the role of classroom climate, AI literacy, and resilience. *Eur. J. Educ.* 60:e12874. 10.1111/ejed.12874

[B98] Wang Y. F. Cionea I. A. (2024). Am I incompetent or just afraid? Competence and apprehension as predictors of intercultural willingness to communicate. *J. Int. Intercult. Commun.* 17 211–228. 10.1080/17513057.2024.2318034

[B99] Weaver C. (2005). Using the Rasch model to develop a measure of second language learners’ willingness to communicate within a language classroom. *J. Appl. Meas.* 6 396–415. 16192663

[B100] Xu Q. Hong H. (2025). Emotional intelligence and language learning performance of ESL learners: mediating effects of L2 grit and L2 motivation. *J. Intell.* 13:146. 10.3390/jintelligence13110146 41295427 PMC12653445

[B101] Yang Y. Liang S. (2025). Classroom social climate and student engagement in English as a foreign language learning: the mediating roles of academic buoyancy and academic emotions. *Asia-Pac. Educ. Res.* 34 1123–1132. 10.1007/s40299-024-00926-2

[B102] Yashima T. MacIntyre P. D. Ikeda M. (2018). Situated willingness to communicate in an L2: interplay of individual characteristics and context. *Lang. Teach. Res.* 22 115–137. 10.1177/1362168816657851

[B103] Ye X. Hu G. (2025). Effects of motivational interventions on EFL learners’ willingness to communicate, self-confidence, and anxiety: an experimental study. *Lang. Teach. Res.* 13621688251367858. 10.1177/13621688251367858

[B104] Yuan L. (2022). Enhancing Chinese EFL students’ grit: the impact of teacher stroke and teacher-student rapport. *Front. Psychol.* 12:823280. 10.3389/fpsyg.2021.823280 35126266 PMC8813776

[B105] Zarrinabadi N. Dehkordi E. S. (2024). The effects of reference of comparison (self-referential vs. normative) and regulatory focus (promotion vs. prevention) feedback on EFL learners’ willingness to communicate. *Lang. Teach. Res.* 28 556–576. 10.1177/13621688211013618

[B106] Zedan R. (2010). New dimensions in the classroom climate. *Learn. Environ. Res.* 13 75–88. 10.1007/s10984-009-9068-5

[B107] Zeng S. Chen Q. (2026). Engagement training effect of EFL learners’ willingness to communicate (WTC) and engagement in classroom activities. *Curr. Psychol.* 45:279. 10.1007/s12144-025-08731-w

[B108] Zhang J. (2023). Modelling the interplay of writing achievement goals and grit in predicting L2 writing achievements. *System* 117:103118. 10.1016/j.system.2023.103118

[B109] Zou M. Noughabi M. A. Peng C. (2025). Modeling the associations between L2 grit, foreign language enjoyment, and student engagement among Chinese EFL English-major learners: a control-value theory perspective. *System* 131:103679. 10.1016/j.system.2025.103679

